# Lubricity potentials of Azadirachta indica (neem) oil and Cyperus esculentus (tiger nut) oil extracts and their blends in machining of mild steel material

**DOI:** 10.1016/j.heliyon.2025.e42059

**Published:** 2025-01-16

**Authors:** Ignatius Echezona Ekengwu, Ikechukwu Geoffrey Okoli, Obiora Clement Okafor, Obiora Nnaemeka Ezenwa, Joseph Chikodili Ogu

**Affiliations:** a Department of Mechanical Engineering, Nnamdi Azikiwe Universit, Awka, Nigeria; b Department of Metalworks Technology Education, Federal College of Education (Technical) Umunze, Nigeria; c Department of Mechanical Engineering, Grundtvig Polytechnic, Oba, Nigeria

## Abstract

Friction amongst the cutting tool and workpiece in metal machining produces heat that reduces tool life and workpiece integrity. Consequently, non-biodegradable soluble mineral oil is predominantly used as a lubricant to enhance machining operations. Nevertheless, recent investigations focus on environmentally friendly biodegradable oils for lubrication. Therefore, this study examines the lubricity potential of neem oil, tiger nut oil, and their blends in machining mild steel. It also evaluates the performance characteristics of individual bio-oils and their blends against conventional soluble mineral oil and dry-drilling methods. Neem and tiger nut oils were extracted using pressing and solvent methods, followed by an analysis of their physiochemical properties. The experimental design utilized the I-Optimal custom design and simplex lattice design (SLD) for the individual and blended oils respectively. Response Surface Methodology (RSM) was applied for optimization, with feed rate, oil type, and spindle speed as independent variables, and cutting temperature, surface finish, depth of cut, chip thickness, chip thickness ratio, cutting speed, and material removal rate as response variables. The optimal cutting conditions were predicted at a spindle speed of 695 rpm, feed rate of approximately 0.4735, and neem oil being the cutting fluid. The predicted response values were cutting temperature - 33.5 °C, surface roughness - 2.65 μm, depth of cut - 41.4825 mm, chip thickness - 0.18951 mm, chip thickness ratio - 269.586, cutting speed - 17.4695 m/min, and material removal rate - 2.38025E-05. Results indicated neem oil surpassed tiger nut oil and conventional oils in minimizing cutting temperatures and enhancing surface quality, achieving a desirability value of 0.85428 under optimal conditions. Moreover, an 80/20 blend of neem and tiger nut oils exhibited improved performance, attaining a desirability value of 0.992, underscoring its potential as an effective cutting fluid. The findings advocate for the use of bio-based cutting fluids in machining operations, indicating environmental and economic advantages while promoting future research into alternative agro-based solutions. However, limitations regarding material applicability and the necessity for further investigation into the micro-structural effects of cutting fluids on diverse engineering materials are acknowledged.

## 1 Introduction

Machining operations of metals and generally engineering materials are associated with heat production that is majorly brought on by frictional interaction between the cutting tool and workpiece interfaces [1]. The generated heat during machining process affects the tool's lifespan and also the surface integrity of the workpiece, and the tool longevity is crucial since significant time is lost whenever a device is replaced or reset [2]. The machining process involves the application of power-driven tools or machines to gradually remove materials from a workpiece to achieve a specific dimension or geometric configuration of the workpiece [1]. Some examples of power-driven equipment are drilling machines, milling machines, shapers, grinders, lathe machines, etc. So, owing to the vast areas of application of these power-driven machines ranging from the industrial sector to small-scale, their usage in machining operations necessitates an in-depth study on the effects of some process parameters – like lubricant type, feed rate, spindle speed, etc. on the temperature, surface finish, depth of cut, chip thickness and cutting speed of the machining process and the workpiece being machined. It is important to note that product quality is hugely dependent on the surface finish [3]. A decrease in surface quality also leads to a reduction of the product's quality in the field of manufacturing, especially engineering. According to Jewo and Ebojoh [3] the surface finish of an engineering material is a factor that is considerably viewed to affect the functioning of a component and possibly its cost. The process parameter assessment is imperative in actualising efficient machining operation and excellent surface finish of the material.

Because of the frictional contact involving the tool and the workpiece interfaces, which causes heat to dissipate during cutting operations, the heat generated by machining operations like drilling, grinding, surfacing, etc. has an impact on the tool life and the product's surface quality. Low product quality and tool deterioration result from improperly dissipating such heat. By applying cutting fluids to the tool and workpiece's machining zone, this issue is minimized. Also, the challenges posed by the mineral oils in areas of non-environmental friendliness, effect on the ozone layer and immense effect on man's health have necessitated the search for better oils that are environmentally friendly, cost-effective, and pose no harmful effect to man's health. Such oil is referred to as vegetable or biodegradable or bio-oil. In addition to their environmental and health safety, they also offer better product quality than the mineral oils. Furthermore, waste to wealth creation is a technology that has empowered so many industries, countries, etc. towards the greater growth of their gross domestic products (GDP) value. The conversion of these agricultural waste products to useful substances employed in the machining operations of engineering materials would greatly impact positively on the GDP of the nation.

The need to mitigate the effects of the generated heat during machining operations so as to achieve good product quality led to the use of non-biodegradable oils which are not environmentally friendly, and also have negative impacts on manufacturing cost and human health [4]. These non-biodegradable oils are also called conventional or soluble oils. Nonconventional machining techniques have proven to be a better choice for machining work materials, according to Kumar and Dvivedi [36]. Also, based on the view of Jabba and Usman [5], the choice of the cutting fluid is just as crucial as the selection of the appropriate machine tools, spindle speed, and feed rate; it is therefore fundamental to access the physiochemical properties and the lubricity potential of the cutting fluids. Sequel to this concern, Lopez et al. [6] stated that the three major effects produced by cutting fluids during machining operations are heat removal, friction prevention on the chip-tool interface, and chips removal. Sokovic and Mijanovic [7] further explained that applying cutting fluid to the machining zone of the tool-workpiece interface results in improved surface finish, quality, and dimensional accuracy. Cutting fluids are lubricants applied to the tool-workpiece interface to reduce frictional effect, absorb the generated heat and provide a good surface finish or generally product quality [1]. Also, as Abou-El-Hossein [8] discusses, applying cutting fluids to the tool-chip interface enables enhanced depth of cut, increased feed rate, increased cutting speeds, longer tool life, reduced surface roughness, improved machining accuracy, and lower energy consumption.

The two broad types of cutting fluids are the mineral oil and vegetable oils. The mineral oils such as the soluble oils pose serious effect to the environment and man, and based on the global requirement for cutting fluids which is solely pinned on factors of renewability, biodegradability, safety and health of man; the vegetable or biodegradable oils are used as their substitute [1]. Mineral oils are derived from crude oil sources. In addition to the mentioned properties of the vegetable oils or bio-lubricants, they also have higher flash/fire points, lower volatility, less oil mist and vapour emissions, and consistent viscosity, all of which contribute to their superior safety [9]. Gawrilow [10] opined that vegetable oils not only offer qualities of environmental safety, renewability, biodegradability, etc. but also provide satisfactory performance in a wide array of applications. Vegetable or biodegradable oils, primarily obtained from the seeds of these biological products, include neem oil, castor oil, tiger nut oil extract, gmelina oil, jatropha oil, honge oil, etc. Because tiger nut and neem oils come from renewable resources, they have a much smaller carbon footprint than petroleum-based lubricants. Additionally, the natural oils' composition offers superior lubrication by minimizing wear and friction—a critical factor in the longevity of machinery [11].

Research efforts have been made towards the study of the performance of these bio-lubricants in the machining operations of mild steel materials. Some of the performance indices used was surface quality, machining temperature, chip thickness ratio, depth of cut, cutting speeds, etc. Eze et al. [1] investigated the effectiveness of castor oil, neem oil, soluble oil, and dry drilling in the drilling of mild steel material; at a spindle speed of 290 rpm and feed rate of 0.0985 mm/rev, castor oil provided the best surface finish for the mild steel material. Eze et al. [4] assessed the efficiency of castor, neem, and Gmelina oils used in the drilling operation of mild steel material; Gmelina oil provided the best surface integrity of the material at a spindle speed of 290 rpm and feed rate of 0.08967 mm/rev with a composite desirability of 1.0. Furthermore, the design and precise selection of a cutting tool for a particular machining application depend heavily on the control and understanding of the chip-tool interface temperature, as stated by Sales et al. [12]. As cutting occurs with a highly negative tool rake angle according to Oliveira et al. [37], Trent [13] clarified that the maximum temperature generated during machining primarily rests on the rake face rather than at the cutting edge of the tool, where the compressive and shear stresses are at their highest. Cutting temperatures, which are established experimentally and utilized as the benchmark for comparison, serve as the foundation for the ranking of cutting fluids. Sales et al. [12] used the thermocouple approach to detect the temperature at the chip-tool interface and investigate the cooling capacity of the cutting fluids. An infrared thermometer connected to a PC and an AC/DC data acquisition board was used to measure the temperature drop of the heated AISI 8640 from 300 °C to room temperature. The tool-chip interface's machining zone was where the cutting fluid was applied. The machining experiments indicated that the application of the fluid with superior cooling ability will not always guarantee reduced chip-tool interface temperature, but also good surface quality. Dhar et al. [14] also conducted experiments to investigate the effect of MQL on cutting temperature, chip, and dimensional accuracy in turning AISI-1040 steel, and a combination of air and soluble oil used as MQL was found to be superior to flooding with soluble oil as a cutting fluid. Furthermore, the tribo-mechanical properties of vegetable-based cutting fluids were studied by Ozcelik [15,16] for sunflower oil, Deshmukh and Hiremath [17] for coconut oil, Srivyas and Charoo [18] for cotton seed oil, Agrawal [28] for castor oil, Babalola [19] for mustard seed oil, and Hassan [20] for palm oil. All of these studies used a variety of metal cutting operations, such as drilling, lathe turning, and milling, and the oils under study showed good anti-wear properties and did not emit any harmful gases into the environment.

In other related studies, Sadiq et al. [21] used a Soxhlet extractor to determine the fatty acids and physicochemical properties of neem seed oil extracts and discovered that linoleic acid predominated; Eziwhuo and Joseph [22] evaluated non-edible seed oil cutting fluid in metal turning operations; Valeru and Suman [23] optimized vegetable oil properties in a machining environment using CFD; Eziwhuo, Ossai, and Alibi [24] examined apricot kernel, avocado, and African pear seed oil as vegetable-based cutting fluids in turning AISI 1020; Lawal and Olalekan [25] investigated the physicochemical properties of melon seed and coconut oil blends and their uses as cutting fluids. The investigation of the lubricity properties of neem and castor oils, along with their mixtures, in the context of machining mild steel materials was conducted by Olawale et al. [26]. The implications of utilizing neem oil as a foundational component in cutting fluids for machining processes were examined in the works of Jabba and Usman [5] and Ademoh, Didam, and Garba [27]. The performance assessment of jatropha and pongamia oil-based environmentally sustainable cutting fluids for the turning of AA6061 was the focus of the research by Jeevan and Jayaram [29]. An inquiry into the performance assessment of African elemi, melon, and African locust bean oil as prospective quenching agents for medium carbon steel was executed by Ibeh et al. [30]. The efficacy of a vegetable oil (Jatropha) based cutting fluid in the machining of steel utilizing minimum quantity lubrication was analyzed by Surase, Pawar, and Bobade [31]. A comparative performance evaluation of neem seed, watermelon seed, and soluble oils as metal cutting fluids was undertaken in the study by Ademoh, Didam, and Garba [27]. The assessment of neem seed oil as a cutting fluid in the orthogonal machining of Aluminum Manganese alloy (AL-MN) during turning operations was executed by Yakubu and Bello [a, b, [32]]. Experimental investigations into cutting parameters during the drilling of mild steel with eco-friendly vegetable oils (neem and Karanja) as cutting fluids were presented by Susmitha, Sharan, and Jyothi [33]. Lastly, the study conducted by Agrawal et al. [28] involved an experimental examination of the wear characteristics of M2 steel utilizing cotton seed oil.

The literature study reveals that while several studies have examined the efficacy of various vegetable oils as cutting fluids, there exist a lack of focused research pertaining specific mixes, particularly those using neem and tiger nut oils. Understanding these bio-oils' practical relevance in industrial settings requires an understanding of how they behave under equivalent machining circumstances, which is something that is not well covered in the majority of current studies. Using neem and tiger nut oil, there is still a lack of knowledge regarding the connection between critical machining parameters (like feed rate, spindle speed, and oil type) and their impact on machining outcomes (like cutting temperature, surface finish, depth of cut, chip thickness, cutting speed, chip thickness ratio, and material removal rate). Because of this, an experiment was conducted to investigates the lubricity potentials of neem/tiger nut seed oil extracts and their blends in machining of mild steel material.

The lubricity and physiochemical properties of neem and tiger nut oils and their blends in the machining of mild steel material were accessed by carrying out performance characteristics of the individual bio-oils and their blends and comparing them to that of a conventional soluble mineral oil and dry-drilling operations. An optimal blend ratio of neem and tiger nut was obtained using an optimization technique where the process variable of feed rate, type of oil and spindle speed were used as the independent variables, while the cutting temperature, surface finish, depth of cut, chip thickness, cutting speed, chip thickness ratio, and material removal rate were used as the response variables. The experimental design for the individual bio-oils and the blend was done with I-Optimal custom design and simplex lattice design (SLD) respectively using design expert software 11.0. In addition, through the assessment of the lubricity potentials of neem/tiger nut seeds oil extracts and their blends as intended in this study, the industries will be impacted on positively as a retrofit to the conventional soluble mineral oil will be determined and knowledge on the effects of process parameters such as oil-type, feed rate, and spindle speed on the response variables-temperature, surface finish, depth of cut, chip thickness, cutting speed, material removal rate and chip thickness ratio would be provided as well.

### 1.1 Research hypothesis

The lubricity of neem oil, tiger nut oil, and their optimal blends will outperform conventional soluble mineral oil in machining operations, resulting in lower cutting temperatures, improved surface finishes, and enhanced material removal rates when machining mild steel.

### 1.2 Specific research objectives

1. To evaluate the lubricity of neem oil, tiger nut oil, and their blends in the machining of mild steel.
2. To compare the performance characteristics of individual bio-oils and their blends against those of conventional soluble mineral oil and dry-drilling operations.
3. To determine the optimal cutting operational conditions (spindle speed, feed rate, and oil type) that yield the best performance metrics (cutting temperature, surface finish, depth of cut, chip thickness, cutting speed, chip thickness ratio, and material removal rate).
4. To apply RSM for the optimization of process variables and to predict the response values for different oil types and blends.
5. To assess the effectiveness of an 80/20 ml mixture of neem and tiger nut oil in comparison to the performance of neem oil alone, determining if the blend offers superior machining characteristics.

The remaining part of this paper consists of four sections. Section 2 introduces the materials used for the study, while section 3 introduces the research method. Section 4 presents results and a discussion. Finally, section 5 covers the conclusion of the study, with contributions and recommendations highlighted.

## 2 Materials used for the study

**Fig. 1:**
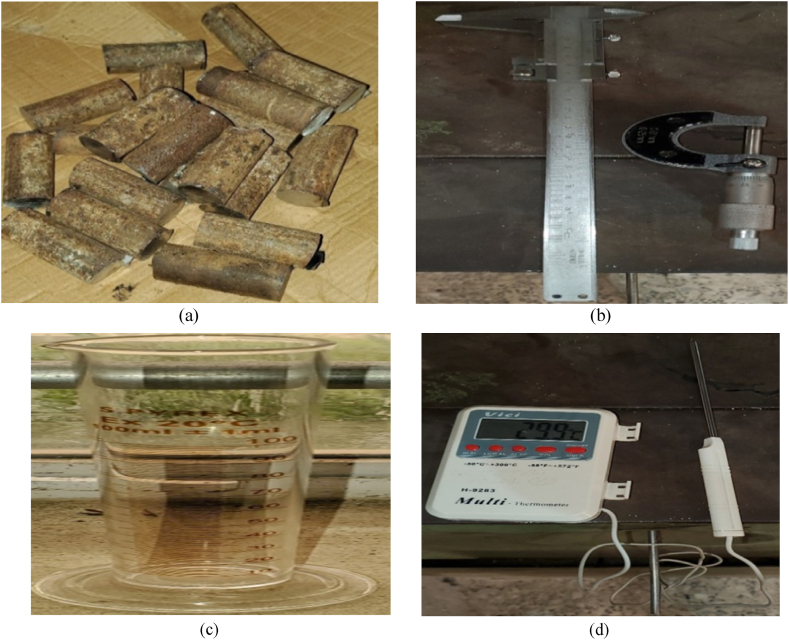
Materials and instruments used in the study: (a) 60 mm cuts of mild steel, (b) vernier caliper and micrometer screw gauge, (c) 100 ml measuring cylinder, (d) handheld digital sensor thermometer.

The materials used in this study include neem seeds, tiger nut seeds, an electric blender, drilling machine, mechanical crushing machine, abrasive papers of grits 220 and 320, 8 mm HSS drill bits, 25 mm diameter mild steel material, digital vernier caliper, micrometer screw gauge, surface roughness tester, digital sensor thermometer, stopwatch, design expert software 11.0, etc. The agricultural seeds of neem and tiger nuts were sourced from Onitsha market and two painters of each were purchased. Fig. 1(a-d) shows some of the experimental images of the materials used in this study. Table 1 shows the specifications of the machines used in this study.

**Table 1:** Specifications/use of the machines/tools used in this study.

	Machine/Tool	Model/Specification	Use
1	Pillar drilling machine	OmmL-12	This machine was used for the drilling operation of the steel material.
2	Electric blender	SC-1589	This was used for grinding the dried tiger nut seeds before the separation of the chaff from the oil.
3	Mechanical crushing machine	GOYUM MK-V-C	This was used for breaking open the neem seeds and also pressed the inner seeds to release its oil content
4	Surface roughness tester	8100	Was used to measure the roughness of the drilled surface after cutting fluid application.
5	Handheld digital sensor thermometer	2-7224-01	Measurement of cutting temperature.
6	Claveland's apparatus	SYD-3536	Was used to determine the flash and fire points of the oil extracts.
7	Viscometer	Model-35	Was used to determine the viscosity of the oil extracts.
8	Calorimeter	SABC-7	Was used to measure the specific gravity of oil extracts.
9	Hydrometer	F-425	Was used to determine the density and specific gravity of the oil extracts.
10	Bruker optics FT-NIR spectrometer	MPA 11	Was used to measure the iodine value of the oil extracts.
11	Vernier caliper and micrometer screw gauge		The vernier calliper was used to measure the depth of cut while the micrometer screw gauge was employed for chips thickness measurement.
12	100 ml measuring cylinder	Pyrez	This was used in measuring the volume of oil to be applied to the drilling zone and also in the blend formulation of the oil extracts.

## 3 Research method

The pressing and solvent method was used to extract the oil content of neem seeds while the blending and separation method was used for the tiger nut seeds. According to Eze et al. [1], the seeds of neem contain about 30%–50 % oil. The neem seeds were first allowed to dry very well under the sun such that the hull could easily be opened for the seeds to be obtained. The seeds were thoroughly cleaned to remove sands, dirt and other unwanted materials. The seeds were crushed using a mechanical pressing machine and neem oil was extracted. The left-over neem cake was further pressed to extract some neem oil from that too. The tiger nut seeds were sun dried very well, after which the seeds were thoroughly washed with water so as to remove dirt and was soaked for about eight (8) hours. The 8 h soaking was to reduce the blending load that would be impacted on the electric blending machine. The soaked tiger nuts were thoroughly blended using an electric blending machine. After the blending operation, the residue or chaff was separated from the oil after 20 min of heating by sieving method. The extracted oils from the seeds are shown in Fig. 3.

### 3.1 Experimental design

After extraction of the oils from the seeds of the agricultural products, an experiment was designed using the design expert software, version 11.0. The design expert software was employed in this study for the use of its specialized two goals – Design of Experiments (DOE) and developing Response Surface Methodology (RSM). For design of the experiment, the I-Optimal Custom design of the RSM variant involving two numeric (Discrete values) factors one categoric (ordinal values) factor at four levels each were employed for the individual oil extract experiment. The runs were set to Lack-of-fits points of one and zero replicate points. Also, the Simplex Lattice Design (SLD) of the mixture variant involving a simplex point of three with zero number of runs to replicate, were employed for the oil extracts blends experiment. The mathematical inequality governing the design was extracted from Jabba and Usman [5] and modified for this experiment is expressed as follows: 0.2mm/rev≤feedrate≤0.5mm/rev and 100rpm≤spindlespeed≤695rpm. A total of sixteen (16) and five (5) runs/simulations were obtained from the experimental design for the individual oils and blends respectively, and was meticulously followed while performing the experiment.

Response surface methodology (RSM) is a group of mathematical and statistical methods for creating empirical model. Optimizing a response (output variable) that is impacted by several independent variables (input variables) is the goal of carefully planned experiments. The response can be graphically shown as contour plots that aid in visualising the response surface's form or in three dimensions. RSM is most widely used in certain situations where a number of input factors may impact a process's quality attribute or performance metric. As a result, the reaction is a quality feature or performance metric. The researcher has control over the input variables, sometimes referred to as independent variables. The field of response surface methodology includes optimization techniques to determine the values of the process variables that yield desired response values, empirical statistical modelling to establish a suitable approximating relationship between the yield and the process variables, and experimental strategy to explore the space of the process or independent variables. In quest of finding the optimum factor combination that would yield the desired response characteristics for neem and tiger nut oils and their blends, I-optimal custom design and simplex lattice mixture design were used respectively. The input variables used were oil type (neem and tiger nut oil), feed rate and spindle speed, while the responses were temperature, surface finish, depth of cut, chip thickness, cutting speed, material removal rate and chip thickness ratio. An additional experiment at the predicted optimal conditions was conducted to validate the model's predictions and observed responses was compared with predicted values.

The mild steel material of diameter 25 mm used for the experiment was thoroughly cleaned to remove dirt and sand particles and was mounted on the table vice of the drilling machine ensuring concentricity between the mild steel and the drill bit to avoid vibrations and lateral/axial gyrations or motions during the machining process. Table 2, Table 3 show the experimental design outputs for the oil extracts and their blends respectively.

**Table 2:** Experimental design for individual oil extracts and soluble oil during dry drilling operations.

Run	Factor 1	Factor 2	Factor 3	Response 1	Response 2	Response 3	Response 4	Response 5	Response 6	Response 7
	A: Feed rate	B: Spindle speed	C: Oil type	Cutting temperature	Surface roughness	Depth of cut	Chip thickness	Chip thickness ratio	Cutting speed	Material removal rate
	mm/rev	rpm	Vol. (ml)	°C	μm	mm	mm		m/mins	$m^{3}$/Mins
1	0.2	100	soluble oil	33.5	2.1	18	0.15	120.0	2.5136	1.0054E-05
2	0.3	205	soluble oil	37.4	2.4	21	0.1	210.0	5.15288	1.5082E-05
3	0.4	345	soluble oil	48	2.8	25	0.59	42.4	8.67192	2.0109E-05
4	0.5	695	Soluble oil	51.2	3.1	32	0.71	45.1	17.46952	2.5136E-05
5	0.2	100	Neem oil	34.8	1.8	20	0.99	20.2	2.5136	1.0054E-05
6	0.3	205	Neem oil	36.2	2.2	24	0.18	133.3	5.15288	1.5082E-05
7	0.4	345	Neem oil	41.9	2.6	31	0.14	221.4	8.67192	2.0109E-05
8	0.5	695	Neem oil	45.2	2.8	48	0.25	192.0	17.46952	2.5136E-05
9	0.2	100	Tiger nut oil	38.1	2.3	15	0.22	68.2	2.5136	1.0054E-05
10	0.3	205	Tiger nut oil	39.8	2.5	18	0.19	94.7	5.15288	1.5082E-05
11	0.4	345	Tiger nut oil	47.7	2.7	30.5	0.175	174.3	8.67192	2.0109E-05
12	0.5	695	Tiger nut oil	52.3	2.9	37	0.182	203.3	17.46952	2.5136E-05
13	0.2	100	Dry drilling	40.1	3.4	12	0.23	52.2	2.5136	1.0054E-05
14	0.3	205	Dry drilling	42.8	3.6	19	0.25	76.0	5.15288	1.5082E-05
15	0.4	345	Dry drilling	49.2	3.7	23	0.29	79.3	8.67192	2.0109E-05
16	0.5	695	Dry drilling	58.8	4	30	0.32	93.8	17.46952	2.5136E-05

**Table 3:** Experimental Design for Blend of the Oil Extracts (performed at a fixed process factors of: feed rate = 0.5m/rev and spindle speed = 695 rpm).

Run	Component 1	Component 2	Response 1	Response 2	Response 3	Response 4	Response 5	Response 6	Response 7
	A:Neem oil	B:Tiger nut oil	Cutting temperature	Surface roughness	Depth of cut	Chip thickness	Chip thickness ratio	Cutting speed	Material removal rate
	Vol. (ml)	Vol. (ml)	°C	μm	mm	mm		m/mins	$m^{3}$/Mins
1	50	50	57.3	3.3	50	0.24	208.33	17.47	2.5136E-05
2	80	20	38.9	2.4	59	0.2	295	17.47	2.5136E-05
3	20	80	52.7	2.8	41	0.195	210.26	17.47	2.5136E-05
4	65	35	48.4	2.5	56	0.17	329.41	17.47	2.5136E-05
5	35	65	50.2	3.1	44	0.18	244.44	17.47	2.5136E-05

**Fig. 2:**
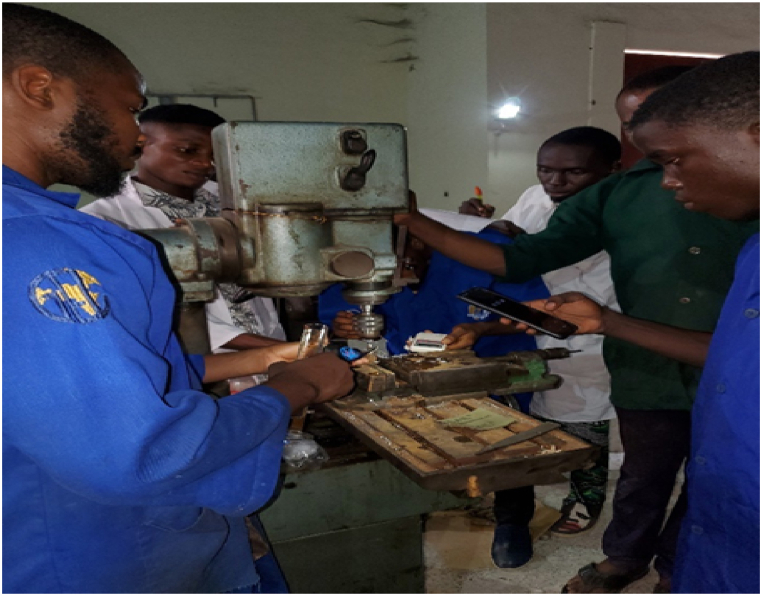
Experimental operations.

The experimental design was meticulously followed for the individual oil extracts and their blends distinctively. For a particular oil type, the specified feed rate and spindle speed was used at three (3) minutes interval to perform drilling operation using 8 mm HSS drill bit, followed by the manual introduction of 10 ml of the cutting fluid at 2 min interval [34]. This process was followed by the response measurements - temperature, surface finish, depth of cut, chip thickness, cutting speed, material removal rate and chip thickness ratio using the appropriate tools. The response characteristics of the conventional soluble mineral oil and dry-drilling operation using the same process factor combinations (feed rate and spindle speed) of the experimental design were also studied and compared to that of neem and tiger nut oil extracts and their blends. The measured responses for the individual oil and blends were optimized in order to get the best process factor combination that would yield the desired or optimum response characteristics. In other words, the optimal blend of the neem and tiger nut mix was obtained through optimization technique. Also, the physiochemical properties of the oil extracts (neem and tiger nut) were ascertained at Springboard laboratory, Awka, Anambra State, Nigeria. The examined physiochemical properties include: flash point, fire point, viscosity, specific heat capacity, density, specific gravity, refractive index and iodine value. The subsequent sections further elucidate on the described methodology of this study. Fig. 2 shows the picture of the experimental operations - drilling, cutting fluid application, temperature measurement, etc.

**Fig. 3:**
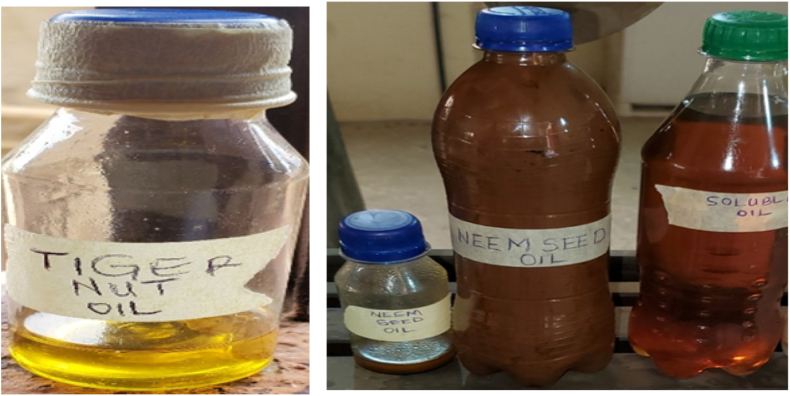
Oil extracts (neem and tiger nut) and conventional oil.

### 3.2 Measurement of the response variables

The cutting temperature, surface roughness, chip thickness and depth of cut were measured using the handheld digital sensor thermometer, surface roughness tester, micrometer screw gauge and vernier caliper respectively. The other responses were computed using the appropriate formulae defined by[35,36,37,38]:

(1) $$\text{Cutting}\;\text{speed}\;\left(V\right)=\frac{\pi \text{DN}}{1000}\;\left(m/\min\right)$$

(2) $$\text{Material}\;\text{removal}\;\text{rate}\;\left(\text{MRR}\right)=\frac{\pi D^{2}}{4}\times F_{m}$$

(3) $$\text{Chip}\;\text{thickness}\;\text{ratio}=\frac{\text{depth}\;\text{of}\;\text{cut}}{\text{chip}\;\text{thickness}}$$

where V is the cutting speed in m/min., D is the diameter of the drill in mm, N is spindle in rpm, and $F_{m}$ is feed rate in m/rev.

Also, one of the popular tools employed to forecast accuracy is the Mean Absolute Percentage Error (MAPE), Kim, S. and Kim, H [38]. Therefore, the mean absolute percentage error (MAPE) applied for the predicted and actual value was calculated by:

(4) $$\text{MAPE}=\frac{1}{N}\sum_{t=1}^{N}|\frac{A_{t}-P_{t}}{A_{t}}|\times 100$$

Where N is the number of data points, $A_{t}$ and $P_{t}$ respectively denotes the experimental and predicted values at the data point *t.* According to Lewis in Mereno et al. [39], the MAPE decision rule states that a MAPE value less than 10 % is a highly accurate prediction, 10%–20 % is a good prediction, 20%–50 % is a reasonable prediction, and avoe 50 % is an inaccurate prediction.

### 3.3 Response optimization

Numerical optimization was employed in optimizing the responses in order to get the best factor combination that would give the optimal desired response characteristics. Also, the optimal mixture blend of neem and tiger oil was obtained through optimization technique. The goal upon which the optimization was based on is delineated in Table 4.

**Table 4:** Optimization goals/criteria.

S/N	Responses	Goal
1	Cutting temperature	Minimize
2	Surface roughness	Minimize
3	Depth of cut	Maximize
4	Chip thickness	0.14–0.43 [43]
5	Cutting speed	Maximize
6	Chip thickness ratio	Maximize
7	Material removal rate	Maximize

### 3.4 Physiochemical studies of neem and tiger nut oil extracts

The physicochemical properties of the oil extracts (neem and tiger nut) were ascertained at Springboard Laboratory, Awka, Anambra State, Nigeria. The examined physiochemical properties include flash point, fire point, viscosity, specific heat capacity, density, specific gravity, refractive index, and iodine value. Table 5 shows the methods and apparatus employed in measuring the physiochemical properties of the oils.

**Table 5:** Physiochemical properties of neem, tiger nut oil extracts, and conventional oil.

S/N	Physiochemical properties	Standard Method	Apparatus
1	Flash point	ASTM D92	Cleveland's apparatus (Open Cup method)
2	Fire point	ASTM D92	Cleveland's apparatus (Open Cup method)
3	Viscosity	ASTM D445	Viscometer
4	Specific heat capacity	ASTM E1269	Calorimeter
5	Specific gravity	AOCS official method 920.212	Hydrometer
6	Density	AOCS official method 920.212	Hydrometer
7	Refractive index	AOCS official method 921.08	refractometer
8	Iodine value	AOCS official method 923.2	Bruker optics FT-NIR spectrometer

∗American Oil Chemist's Society (AOCS).

∗American society for testing and material (ASTM).

## 4 Results and discussion

In this section, the results obtained from the laboratory experiment and software simulation are presented in subsections 4.1 to 4.4.

### 4.1 Results of the physiochemical properties of the oil extracts –neem and tiger nut

Table 6 delineates the measured physiochemical properties of neem oil extract, tiger nut oil extract, and the conventional soluble oil. Table 6 shows the methods employed in the measurement of the physiochemical properties of the oils.

**Table 6:** Physiochemical properties of neem, tiger nut oil extracts, and conventional oil.

S/N	Physiochemical properties	Units	Neem oil	Tiger nut oil	Conventional oil	80/20 blend
1	Flash point	Co	249	165	204	231.2
2	Fire point	Co	287	174	371	265.3
3	Viscosity	Pa.s at 34 Co	0.7572	0.7240	0.9691	0.7511
4	Specific heat capacity	KJ/kg.k	1.6819	1.4980	1.828	1.6485
5	Density	g/ml	0.9621	0.9170	0.9970	0.9536
6	Specific gravity	–	0.9418	0.9213	0.9865	0.9381
7	Refractive index	–	1.4582	1.4680	1.4117	1.4601
8	Iodine value	g/100 g	100.28	78.26	116.33	95.93

Physiochemical properties of the soluble oil is obtained from the product chart.

From Table 6, neem oil had higher flash point and fire point temperatures, viscosity, specific heat capacity, density, specific gravity, and iodine values when compared to tiger nut oil. However, Tiger nut oil had a higher refractive index value than neem oil. From the physiochemical assessment of the two oil extracts neem oil appeared to possess superior cutting fluid properties [5] than tiger nut oil. According to Utono et al. [40], seasonal variations, genetic differences, geographical origin, plant segments, growth stages, and postharvest processing methods significantly influence the superior chemical composition of neem oil. In agreement to this, Ismaila et al. [21], stated that neem oil exhibits superior cutting fluid properties compared to tiger nut oil, benefiting from higher viscosity and thermal stability, making it preferable for machining applications. Also, comparing the physiochemical properties of conventional oil to neem oil, the property difference between the two are significant for applications involving heat, which explains the choice of neem oil when deployed in a cutting zone. Neem oil's reduced viscosity and stable physicochemical qualities under heat make it suitable for cutting applications, improving handling and performance when compared to conventional oils [21]. The physicochemical properties of the 80/20 blend show that it combines the advantages of neem oil's lubrication and thermal stability with the smoother flow characteristics of tiger nut oil, making it a versatile and effective option for machining applications.

### 4.2 Effects of cutting fluid application on the response variables at varying feed rates and spindle speed

**Fig. 4:**
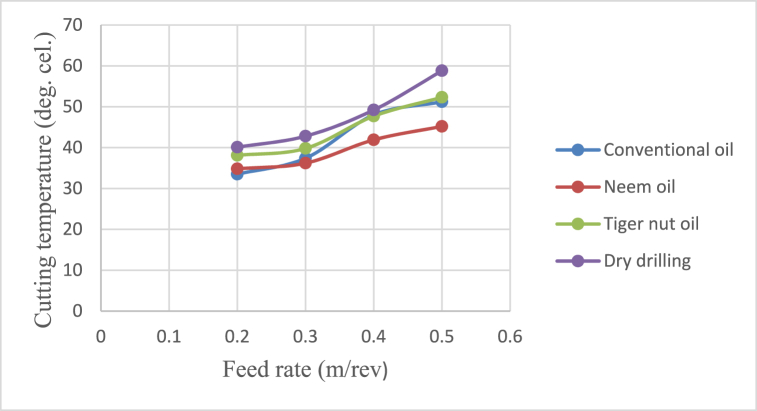
Effect of cutting fluid application on the cutting temperature of mild steel material at varying feed rates.

**Fig. 5:**
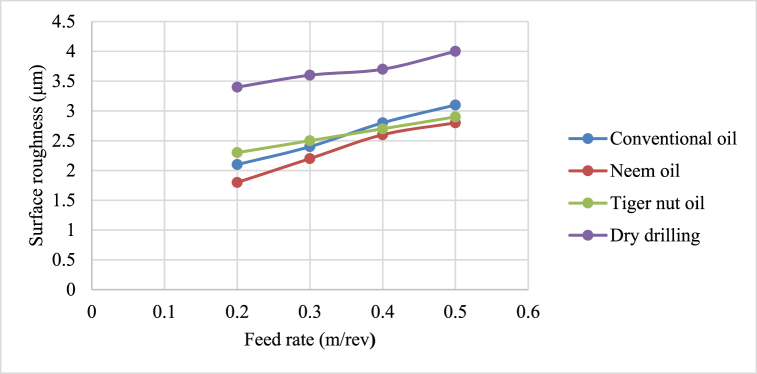
Effect of cutting fluid application on surface roughness at varying feed rates.

**Fig. 6:**
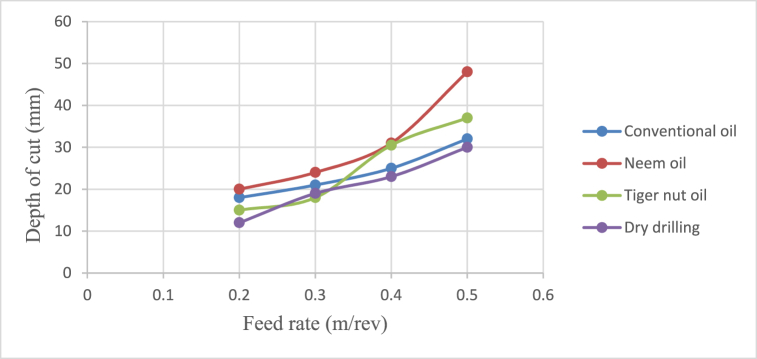
Effect of cutting fluid application on the depth of cut at varying feed rate.

**Fig. 7:**
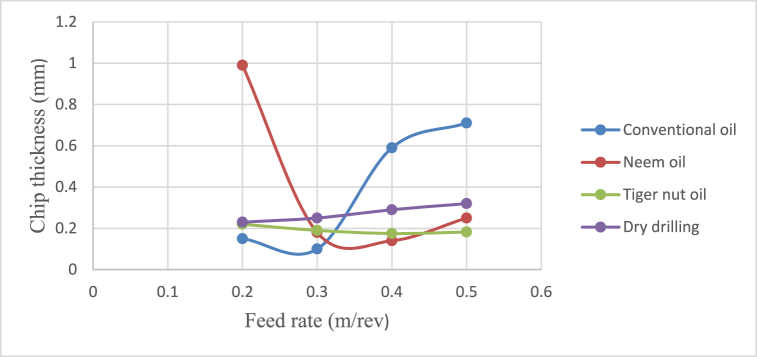
Effect of cutting fluids on the chip thickness using varying feed rates.

Fig. 4, Fig. 5, Fig. 6, Fig. 7 show the effect of cutting fluid on the cutting temperature of mild steel material, the effect of oil application on the surface roughness of the workpiece, effect of cutting fluid application on the depth of cut, effect of cutting fluids on the chip thickness at varying feed rates using the conventional oil, neem oil, tiger nut oil and the dry drilling operation.

From Fig. 4, neem oil appeared to reduce the cutting temperature generated by the frictional heat at the cutting zone of the tool and workpiece material than the other oils applied. This may be explained by the improved lubrication and high thermal capacity, which lower friction between the tool and the workpiece and increase cutting depth and efficiency [41]. The conventional oil reduced the cutting temperature more than tiger nut oil. Also, dry drilling operation could be observed to have higher temperature compared to oil-applied operations. Additionally, as the cutting operation's feed rate increases, the cutting temperature rises as well, significantly affecting the tool life [35]. This behaviour resulted from the increase in the generated heat by friction during the tool's linear cutting action [41]. In Fig. 5, neem oil had the lowest surface roughness (SR) value of 1.8 μm at a temperature of 34.8 °C, feed rate of 0.2m/rev and spindle speed of 100 rpm. This was followed by conventional oil which had a SR value of 2.1 μm under the same conditions and then tiger nut oil with a SR value of 2.3 μm. the low values of the surface roughness conform with Ademoh et al. [27], who stated that the acceptable standard for turned and machined surfaces is maximum of 25 μm. The low value of SR obtained by using neem oil as the cutting fluid resulted from its ability to decrease cutting temperature throughout machining operations by absorbing the generated heat. Heat is known to affect the SR of engineering materials. It can be seen from Fig. 6, neem oil had the highest depth of cut value of 48 mm followed by tiger nut oil- 37 mm, and then the conventional oil- 32 mm. Drilling operation at no-fluid application had the lowest value of 30 mm. Heat affects the cutting efficiency of a cutting tool drill bit, and as such, if the quality of the cutting fluid applied at the cutting interface between the tool and the workpiece is not good, the cutting ability of the tool is reduced and this would consequently affect the cut-depth of the tool. Therefore, the observed value of cut-depth using neem oil suggests that it is of better quality and more effective than the rest of the oils that were deployed to the cutting zone which is also attributed to the neem oil's effective heat absorption that enhances the cutting efficiency and resulting in a greater cut-depth compared to other oils [33]. From Fig. 7, the thickness of the chips could be observed to vary significantly as the feed rate increases from 0.2m/rev to 0.5m/rev. Therefore, the type of cutting fluid and the feed rate used in a cutting operation affect the thickness of the chip produced.

**Fig. 8:**
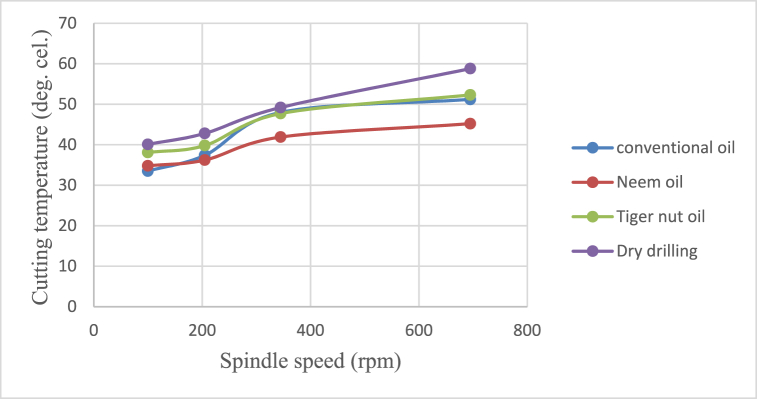
Effect of cutting fluid application on cutting temperature at varying spindle speed.

**Fig. 9:**
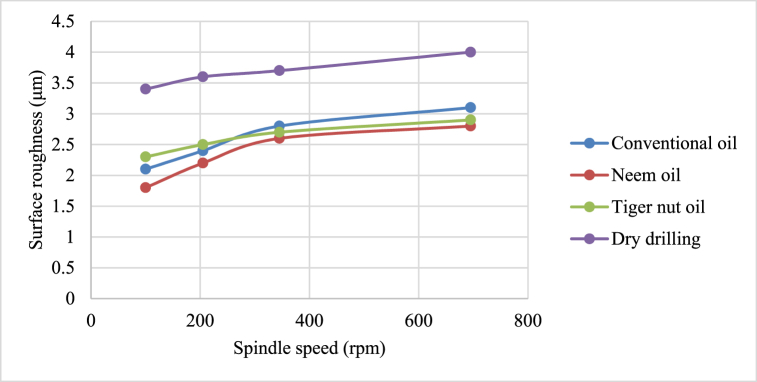
Effect of cutting temperature on surface roughness at varying spindle speeds.

**Fig. 10:**
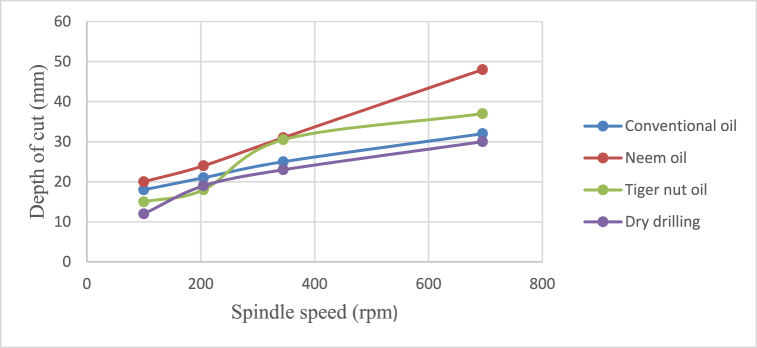
Effect of cutting fluid application on depth of cut at varying spindle speed.

**Fig. 11:**
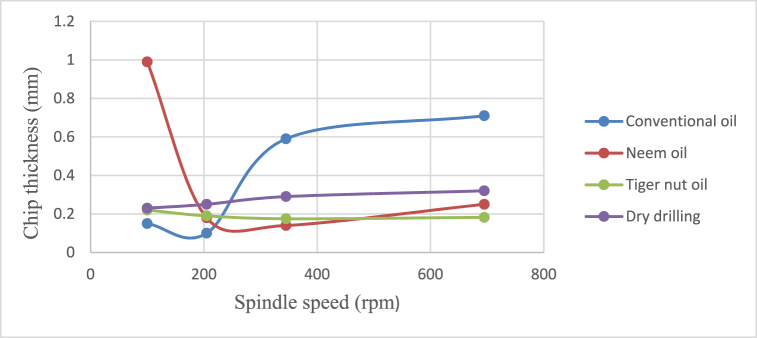
Effect of cutting fluid application on chip thickness at varying spindle speed.

The effects of cutting fluid application on cutting temperature, cutting temperature effect on surface roughness, cutting fluid application effect on depth of cut, and cutting fluid application effect on chip thickness at different spindle speeds using conventional oil, neem oil, tiger nut oil, and dry drilling operation are shown in Fig. 8, Fig. 9, Fig. 10, Fig. 11.

From Fig. 8, Fig. 9, Fig. 10, Fig. 11, it could be observed that as the spindle speed increases from 100 to 695 rpm, the cutting temperature, surface roughness and depth of cut increase as well for all the cutting fluids applied, with neem oil having the best values for all the response variables - cutting temperature, surface roughness, depth of cut and chip thickness; followed by the conventional oil and then tiger nut oil. These best values of neem oil for the response variables are attributed to neem oils' superior lubricating properties which effectively reduce friction and heat generation during machining [41]. Drilling operation without cutting fluid application could be observed to yield poor workpiece quality [2,42] because no fluid was applied to absorb the frictionally generated heat of the drilling operation. Therefore, spindle speed and cutting fluid affect the product quality of mild steel and generally, engineering materials.

### 4.3 Optimization using response surface methodology (RSM) for individual oil

Fig. 12, Fig. 13, Fig. 14, Fig. 15, Fig. 16, Fig. 17, Fig. 18, Fig. 19, Fig. 20 delineate the contour plots of the desirability values and all the response variables used in the numerical optimization operation.

**Fig. 12:**
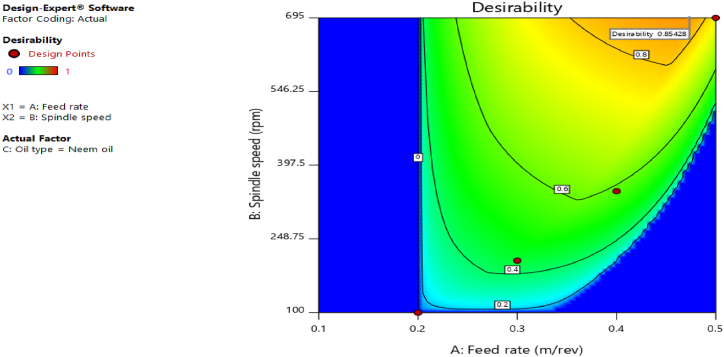
Desirability plot.

**Fig. 13:**
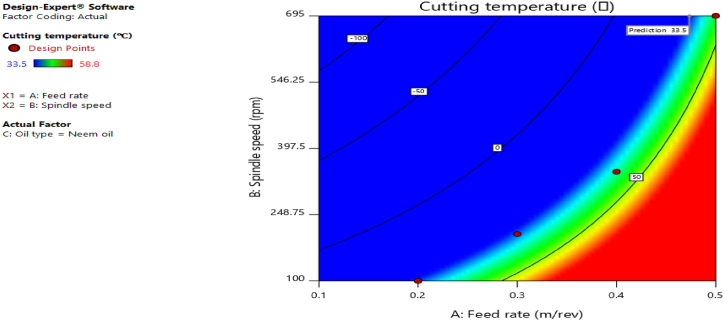
Contour plot of spindle speed against feed rate for cutting temperature.

**Fig. 14:**
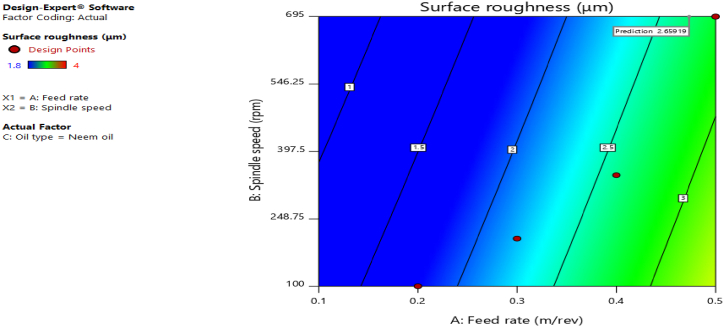
Contour plot of spindle speed against feed rate for surface roughness.

**Fig. 15:**
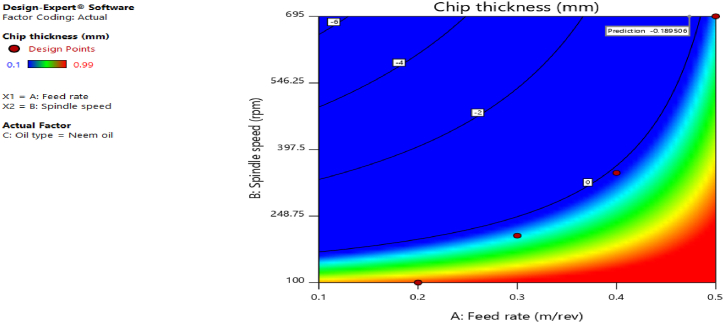
Contour plot of spindle speed against feed rate for chip thickness.

**Fig. 16:**
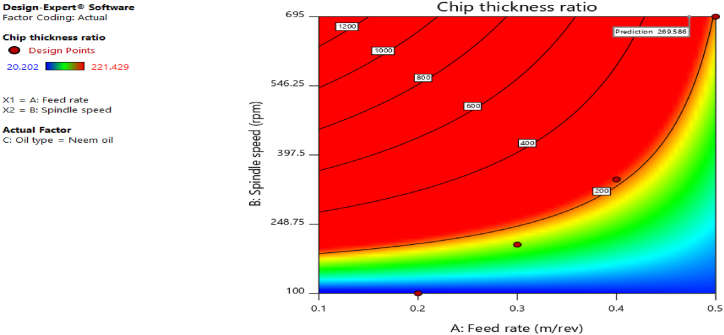
Contour plot of spindle speed against feed rate for chip thickness ratio.

**Fig. 17:**
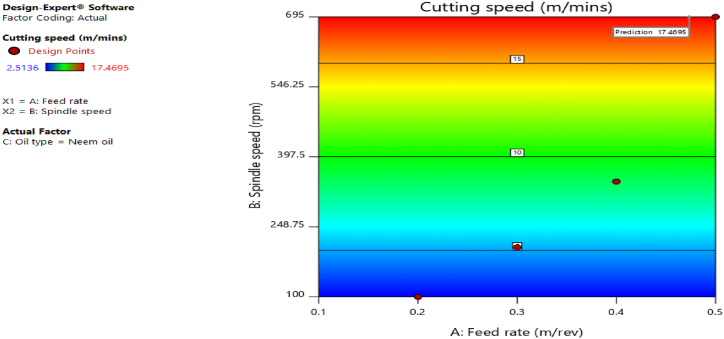
Contour plot of spindle speed against feed rate for cutting speed.

**Fig. 18:**
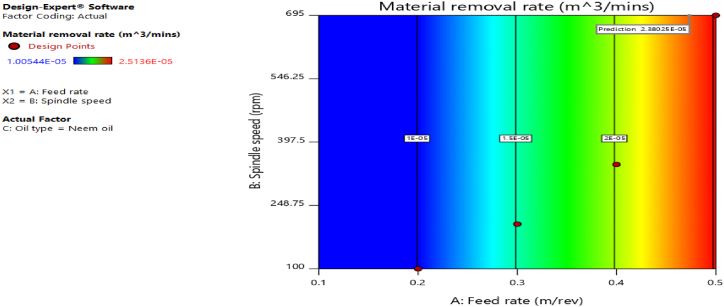
Contour plot of spindle speed against feed rate for material removal rate.

**Fig. 19:**
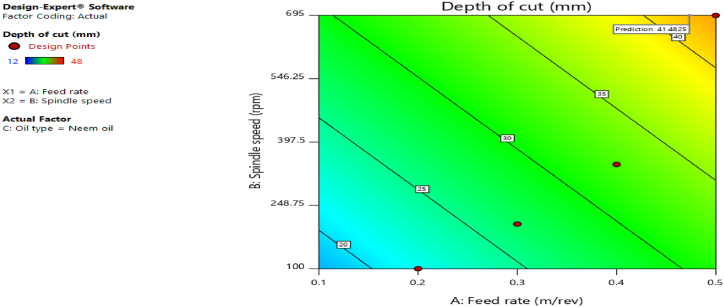
Contour plot of spindle speed against feed rate for depth of cut.

**Fig. 20:**
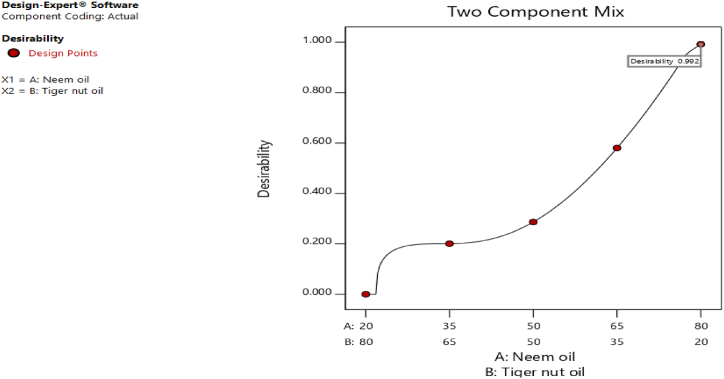
Desirability plot.

From Fig. 12, a desirability value of 0.85428 was obtained as the optimal solution which suggest that neem oil is the best cutting fluid for the application and conditions for which it was deployed. At the optimal design point as shown in the desirability plot of Fig. 12, the best cutting operational condition was predicted to be at a spindle speed of 695 rpm, feed rate of approximately- 0.4735 and neem oil as the cutting fluid. At this optimal design point, the predicted response values using the models were shown in Fig. 13, Fig. 14, Fig. 15, Fig. 16, Fig. 17, Fig. 18, Fig. 19. The response values at the optimal point were; cutting temperature - 33.5 °C, surface roughness - 2.65 μ m, depth of cut - 41.4825 mm, chip thickness - −0.18951 (the negative value describes how small the chip thickness is), chip thickness ratio- 269.586, cutting speed - 17.4695 m/min and material removal rate - 2.38025E-05. Table 7 delineates the statistics of this optimal design point.

**Table 7:** Summary statistics of the optimal design variables (Two-sided Confidence = 95 %).

Solution 1 of 100 Response	Predicted Mean	Predicted Median	Std Dev	n	SE Pred	95 % PI low	95 % PI high
Cutting temperature	33.5	33.5	0.774379	1	2.39744	25.8703	41.1297
Surface roughness	2.65919	2.65919	0.0379652	1	0.117538	2.28513	3.03325
Depth of cut	41.4825	41.4825	3.21312	1	3.99745	32.5756	50.3893
Chip thickness	−0.189506	−0.189506	0.14012	1	0.433804	−1.57006	1.19105
Chip thickness ratio	269.586	269.586	55.8482	1	172.903	−280.669	819.84
Cutting speed∗	17.4695	17.4695	0	1	0	17.4695	17.4695
Material removal rate∗	2.38025E-05	2.38025E-05	0	1	0	2.38025E-05	2.38025E-05

### 4.4 Optimization of the blend using RSM

**Fig. 21:**
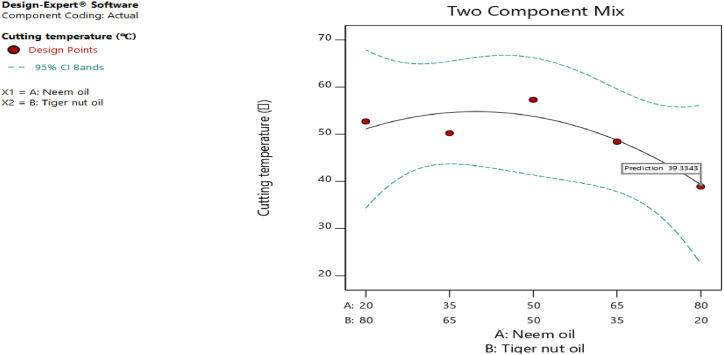
Plot of cutting temperature against the mixture ratios.

**Fig. 22:**
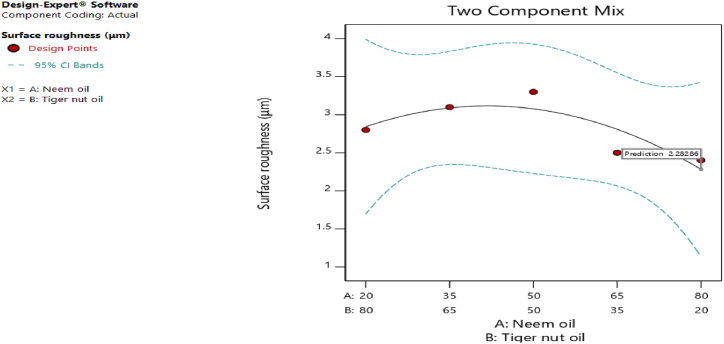
Plot of surface roughness against mixture composition ratio.

**Fig. 23:**
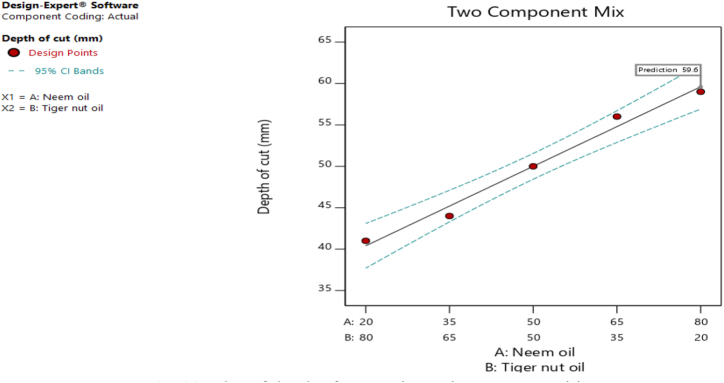
Plot of depth of cut against mixture compositions.

**Fig. 24:**
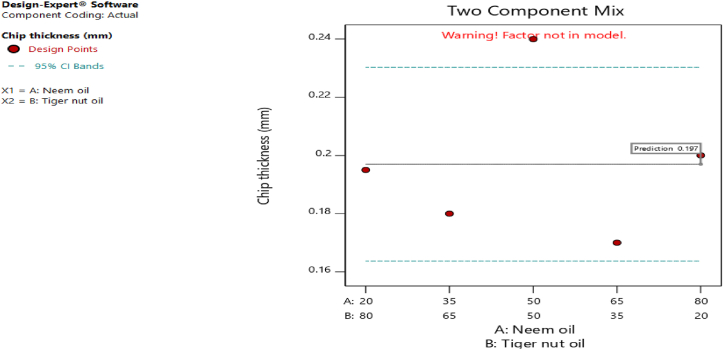
Plot of chip thickness against mixture compositions.

**Fig. 25:**
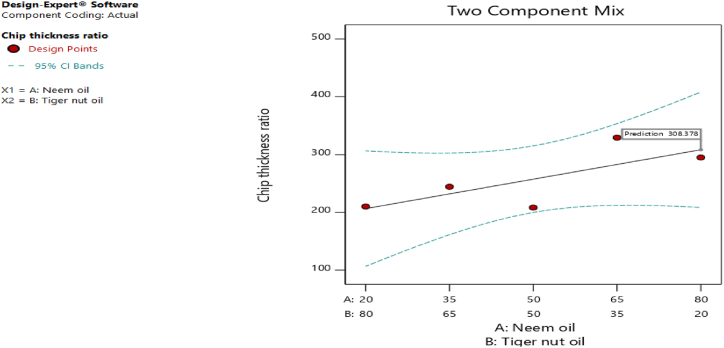
Plot of chip thickness ratio against mixture compositions.

Fig. 20, Fig. 21, Fig. 22, Fig. 23, Fig. 24, Fig. 25 clearly depict the numerical optimization graphs for the selection of the optimal factors combination that would yield the best response characteristics. The experiments for the mixture of neem and tiger nut oils were done at a constant feed rate of 0.5m/rev and spindle speed of 695 rpm. Hence, the cutting speed and material removal rate had constant values of 17.47 m/min and 2.5136E-05 respectively. Fig. 20 revealed the desirability plot, while Fig. 21, Fig. 22, Fig. 23, Fig. 24, Fig. 25 showed the response predictions at the obtained optimal design point using the generated models.

Fig. 20 clearly showed the optimal design point at a desirability value of 0.992 and mixture compositional ratio of neem/tiger nut oils - 80/20 ml. At this optimal design point, the predicted values of the response variables using the generated models are shown in Fig. 21, Fig. 22, Fig. 23, Fig. 24, Fig. 25. From Fig. 21, Fig. 22, Fig. 23, Fig. 24, Fig. 25, a cutting temperature of 39.3342 °C, surface roughness value of 2.28286 μm, depth of cut- 59.6 mm, chip thickness- 0.197 and chip thickness ratio of 308.378 were obtained at the optimal design point. By comparing these optimal response characteristics of 80/20 ml neem/tiger nut oil mixture with that obtained in subsection 4.3 where neem oil was gotten as the optimal fluid, the mixture of 80/20 ml neem/tiger nut oils appeared to be more effective than just neem oil application. Table 8 shows the optimal performance values for neem oil and 80/20 ml of neem/tiger nut oil blend. From the table,

**Table 8:** Optimal performance study of neem oil and 80/20 ml neem/tiger nut oil.

S/N	Optimal measuring tool	Neem oil	Predicted values 80/20 ml neem/tiger nut oil
1	Desirability	0.85428	0.992
2	Cutting temperature	33.5 °C	39.3342 °C
3	Surface roughness	2.66 μ m	2.28286 μ m
4	Depth of cut	41.4825 mm	59.6 mm
5	Chip thickness	0.18951 mm	0.197 mm
6	Chip thickness ratio	269.586	308.378

In the comparative analysis of the Response Surface Methodology (RSM) results for Neem oil and an 80/20 neem/tiger nut oil mixture, the latter demonstrates a markedly elevated desirability value of 0.992, suggesting that the mixture exhibits a greater efficacy than Neem oil utilized in isolation. However, it is noteworthy that the marginal increase in feed rate associated with the mixture may have played a role in enhancing performance, as both experimental configurations were maintained at an identical spindle speed of 965 rpm, thereby facilitating a direct assessment of their relative effectiveness under uniform rotational conditions. The cutting temperature recorded for neem oil was 33.5 °C, indicating a superior cooling effect relative to the mixture, which exhibited a higher temperature of 39.3342 °C; this discrepancy suggests that the blend may have induced additional thermal generation, potentially attributed to increased friction and augmented cutting efficiency. This phenomenon is corroborated by the observed reduction in surface roughness for the blend, which measured 2.28 μm in contrast to the 2.65 μm observed for neem oil alone. Furthermore, the depth of cut achieved with the 80/20 neem/tiger nut oil mixture surpasses that of neem oil by 18.1175 mm, implying that the mixture facilitates deeper cuts, thereby indicating a propensity for enhanced productivity through substantial material removal in a singular operational process. Additionally, the increased chip thickness and chip thickness ratio of 0.197 mm and 308.378, respectively, further illustrate that the 80/20 neem/tiger nut oil blend significantly enhances efficiency in material removal, accompanied by improved chip formation and management.

#### 4.4.1 Mean absolute percentage error analysis (MAPE)

This statistical metric serves to evaluate the precision of the anticipated values derived from an experimental study. It articulates the mean of the percentage discrepancies observed between the predicted and empirical values. The absolute percentage error was utilized to ascertain the error associated with the specific response variable, whereas the mean absolute percentage error delineates the overall error present within the experimental framework. Analysis presented in Table 9 indicates that the assessment of absolute percentage errors reveals a remarkably high degree of accuracy in the predictions pertaining to all response variables. The findings further imply that, despite the high accuracy of the predictions, further refinement of the predictions concerning surface roughness and chip thickness is warranted, as their associated error rates are comparatively higher than those of cutting temperature, depth of cut, and chip thickness. In summary, the mean absolute percentage error of approximately 2.62 % indicates that the deviation from the predictions is relatively minimal, thereby suggesting that the predictive model exhibits a satisfactory performance across the various measurements.

**Table 9:** Error analysis for the optimum experimental and predicted values of 80/20 ml neem/tiger nut oil.

S/N	Optimal measuring tool	Experimental values 80/20 ml neem/tiger nut oil	Predicted values 80/20 ml neem/tiger nut oil	Mean Absolute Percentage Error (%)
1	Cutting temperature	38.9 °C	39.3342 °C	1.10
2	Surface roughness	2.4 μ m	2.28286 μ m	5.14
3	Depth of cut	59.0 mm	59.6 mm	1.01
4	Chip thickness	0.2 mm	0.197 mm	1.52
5	Chip thickness ratio	295.0	308.378	4.34

## 5 Conclusion

The utilization of cutting fluids at the tool-workpiece interface significantly enhances surface quality and reduces heat from friction. Physicochemical analysis indicated that neem oil exhibits superior properties compared to tiger nut oil and is comparable to conventional oils. Furthermore, neem oil notably decreased cutting temperatures relative to tiger nut and conventional oils. Additionally, neem oil produced optimal response characteristics as per numerical optimization results (RSM). The RSM yielded a desirability value of 0.85428, with optimal cutting conditions identified as a spindle speed of 695 rpm and a feed rate of approximately 0.4735, utilizing neem oil. At these optimal conditions, predicted response values included: cutting temperature - 33.5 °C, surface roughness - 2.65 μm, depth of cut - 41.4825 mm, chip thickness - −0.18951, chip thickness ratio - 269.586, cutting speed - 17.4695 m/min, and material removal rate - 2.38025E-05. Moreover, RSM optimization of the neem and tiger nut oil mixture achieved a desirability value of 0.992, with predicted values of cutting temperature - 39.3342 °C, surface roughness - 2.28286 μm, depth of cut - 59.6 mm, chip thickness - 0.197, and chip thickness ratio - 308.378. Comparative analysis revealed that the 80/20 ml neem/tiger nut oil mixture outperformed neem oil alone in optimal response characteristics. This research contributes to existing knowledge by evaluating the operational characteristics of neem and tiger nut oils against conventional oils, highlighting neem oil as the superior choice, and demonstrating that the optimal 80/20 neem/tiger nut oil blend is more effective than neem oil alone. The findings advocate for the use of the neem/tiger nut oil blend as an effective alternative in machining, potentially enhancing surface quality and mitigating thermal stress on tools and workpieces. Additionally, employing agro-based cutting fluids like neem and tiger nut oils could lower costs associated with conventional cutting oils and foster sustainability in manufacturing. Transitioning to bio-based cutting fluids may reduce the environmental impact of machining operations, supporting green manufacturing objectives. Other agro products should be investigated for cutting fluid research and compared with this study's findings. Dates should be incorporated with tiger nuts prior to oil extraction, and operational characteristics should be analyzed relative to this study. Furthermore, this research exclusively focused on mild steel, which restricts the applicability of the results to other materials. The micro-structural properties of the machined material were not assessed, hindering the comprehension of the impact of cutting fluids on material characteristics. Additionally, the influence of the cutting fluids on the material's structural integrity and the molecular interactions with mild steel were not examined in this research.

## CRediT authorship contribution statement

**Ignatius Echezona Ekengwu:** Conceptualization. **Ikechukwu Geoffrey Okoli:** Writing – original draft, Software, Formal analysis, Validation. **Obiora Clement Okafor:** Writing – review & editing. **Obiora Nnaemeka Ezenwa:** Investigation. **Joseph Chikodili Ogu:** Resources, Data curation.

## Data availability

Data supporting this study will be made available on request.

## Declaration of Competing Interest

The authors declare that they have no known competing financial interests or personal relationships that could have appeared to influence the work reported in this paper.
